# Anticancer Strategy Targeting Cell Death Regulators: Switching the Mechanism of Anticancer Floxuridine-Induced Cell Death from Necrosis to Apoptosis

**DOI:** 10.3390/ijms21165876

**Published:** 2020-08-16

**Authors:** Akira Sato, Akiko Hiramoto, Hye-Sook Kim, Yusuke Wataya

**Affiliations:** 1 Department of Biochemistry and Molecular Biology, Faculty of Pharmaceutical Sciences, Tokyo University of Science, 2641 Yamazaki, Noda, Chiba 278-8510, Japan; 2 Division of International Infectious Disease Control, Faculty of Pharmaceutical Sciences, Okayama University, 1-1-1 Tsushima-naka, Kita-ku, Okayama 700-8530, Japan; akihira1076@gmail.com (A.H.); hskim@cc.okayama-u.ac.jp (H.-S.K.); twksjo@yahoo.co.jp (Y.W.)

**Keywords:** necrosis, apoptosis, transcriptome analysis, proteome analysis, microRNA, cell death regulator

## Abstract

Cell death can be broadly characterized as either necrosis or apoptosis, depending on the morphological and biochemical features of the cell itself. We have previously reported that the treatment of mouse mammary carcinoma FM3A cells with the anticancer drug floxuridine (FUdR) induces necrosis in the original clone F28-7 but apoptosis in the variant F28-7-A. We have identified regulators, including heat shock protein 90, lamin-B1, cytokeratin-19, and activating transcription factor 3, of cell death mechanisms by using comprehensive gene and protein expression analyses and a phenotype-screening approach. We also observed that the individual inhibition or knockdown of the identified regulators in F28-7 results in a shift from necrotic to apoptotic morphology. Furthermore, we investigated microRNA (miRNA, miR) expression profiles in sister cell strains F28-7 and F28-7-A using miRNA microarray analyses. We found that several unique miRNAs, miR-351-5p and miR-743a-3p, were expressed at higher levels in F28-7-A than in F28-7. Higher expression of these miRNAs in F28-7 induced by transfecting miR mimics resulted in a switch in the mode of cell death from necrosis to apoptosis. Our findings suggest that the identified cell death regulators may play key roles in the decision of cell death mechanism: necrosis or apoptosis.

## 1. Introduction

The cell death mechanisms have become an extremely important research subject for understanding the cell survival and cell proliferation of cancer. Cell death can be broadly characterized as either necrotic or apoptotic, depending on the morphological, and biochemical features of the cell itself [1,2,3]. Previous studies have reported various types of cell death mode, including apoptosis, necroptosis, pathanatos, programmed necrosis, and necrosis [1,2,3,4,5]. Apoptotic cells shrink, form cell fragments called apoptotic bodies, and are phagocytosed by phagocytes in vivo. Cancer cells regress or disappear following apoptosis induced by treatment with anticancer drugs or radiation. Conversely, necrosis causes the swelling of cells and inflammation of neighboring cells, leading to the leakage of cell contents into the extracellular space. Necrosis is recognized as a side effect of cancer treatments with anticancer agents and radiation. In addition, necrosis in the tumor microenvironment generally contributes to resistance to treatment with anticancer drugs and radiation and causes hemorrhage and edema from the tumor tissue [6,7,8]. The incidence of necrosis has become an important clinical problem in cancer treatment.

We hypothesize that there is a mechanistic switch that determines whether a particular stimulus induces cell death via necrosis or apoptosis. We have established a cell model in which either necrosis or apoptosis can be induced via treatment with identical concentrations of the same drug. Moreover, we have compiled available research on the mechanisms of cell death and switching between necrosis and apoptosis. In this review, we outline the characteristics of these two types of cell death, the cellular models to study the mechanism of switching between them, the search for molecular regulators and their functional analysis, and the strategies targeting these regulators to treat cancers.

## 2. Characteristics of the Cell Death-Switching Model System

**Figure 1:**
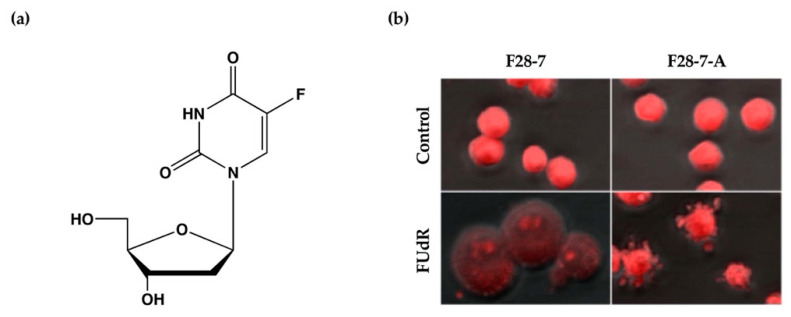
(**a**) Chemical structure of anticancer drug floxuridine (5-Fluoro-2′-deoxyuridine, FUdR). (**b**) Cell death morphological changes induced by FUdR in F28-7 and F28-7-A cell lines. FUdR induces necrosis in F28-7 and apoptosis in F28-7-A. Control, no treatment; FUdR, cells were treated with 1 μM FUdR for 21 h. Figure 1b modified from Ref. [9].

We have previously established a mouse mammary tumor FM3A cell line F28-7 that undergoes necrosis when treated with the anticancer drug floxuridine (FUdR) (1 μM) (Figure 1a) and a sub-clone variant F28-7-A cell line that spontaneously undergoes apoptosis when treated with the same concentration of FUdR [9]. FUdR suppresses DNA synthesis by inhibiting thymidylate synthase [10,11]. The concentration at which 50% of cells were effected (EC_50_) was 1 nM in F28-7 and F28-7-A cells [9,12]. Originally, FM3A cells underwent necrosis following FUdR treatment, but when a subculture was continued, cells with different properties emerged, including those that underwent apoptosis upon FUdR treatment. We established two sub-clone variants of F28-7 cells: one that underwent necrosis and another that underwent apoptosis (F28-7-A cells) by limiting dilution [9]. Figure 1b presents the morphology of cell death of F28-7 and F28-7-A cells following treatment with FUdR. In F28-7 cells, the swelling of nuclei and cells characteristic of necrosis was observed following FUdR treatment. Conversely, in F28-7-A cells, apoptotic bodies were found. In addition, DNA fragmentation of oligonucleosome units specific to apoptosis was also detected. Furthermore, the release of cytochrome c from the mitochondria was also confirmed. From the above, it was concluded that F28-7 cells undergo necrosis and F28-7-A cells undergo apoptosis following the action of FUdR. Table 1 presents the characteristics of necrotic and apoptotic cell death in these two cell sublines. The establishment of a unique cell line that shifts the mechanism of cell death without artificial gene manipulation is extremely rare, suggesting that there is a mechanism by which cell death switches from necrosis to apoptosis. Moreover, by using these two cell lines, it is possible to screen for cell death regulators that determine the pathway of necrosis or apoptosis in response to several stimuli and to analyze the cell death-switching mechanism.

## 3. Functional Analysis of Candidate Molecular Switches Regulating Necrosis and Apoptosis

We investigated the cell death regulators of two types of cancer cell death, necrosis and apoptosis, using the F28-7 and F28-7-A cell death models. First, the regulators that differ in mRNA and protein expression levels in these cell models were analyzed via comprehensive transcriptomic and proteomic analysis. We show that the activating transcription factor 3 (*Atf3*) was highly expressed in cells that underwent FUdR-induced necrosis (F28-7 cells), compared with those that underwent FUdR-induced apoptosis (F28-7-A cells), by transcriptomic analysis using a cDNA microarray [13,14]. In addition, we investigated the comprehensive protein expression levels of cell death regulators by proteomic analysis using two-dimensional electrophoresis, matrix-assisted laser desorption/ionization time-of-flight mass spectrometry (MALDI-TOF-MS/MS), and nano-liquid chromatography–tandem mass spectrometry (LC-MS/MS) [15]. We have shown that at the untreated and FUdR-treated stages, the levels of the nuclear and cytoplasmic intermediate filament proteins lamin-B1 and cytokeratin-19 are higher in F28-7 than in F28-7-A cells [15]. Functional analysis of the cell death regulator candidates (ATF3, lamin-B1, and cytokeratin-19) was conducted using small interfering RNA (siRNA) knockdown of each candidate gene, followed by phenotypic screening of the mode of FUdR-induced cell death [14,15,16]. ATF3, lamin-B1, and cytokeratin-19 were all expressed at higher levels in F28-7 (originally FUdR-induced, necrosis-fated cells) than in F28-7-A cells (apoptosis-fated cells). Furthermore, we found that the individual knockdown of these candidates in FUdR-treated, necrosis-fated cells resulted in a shift from necrotic to apoptotic cell death. These results suggest that the transcription factor ATF3, the nuclear scaffold lamin-B1, and the cytoplasmic intermediate filament cytokeratin-19 are all potential regulators of the mode of cell death [13,14,15,16].

ATF3 is a member of the ATF/cyclic AMP response element binding (ATP/CREB) family of transcription factors and is induced by DNA damage and various oncogenic stimuli [17]. ATF3 is known to function as a transcription factor to regulate gene expression, thereby contributing to cellular responses to oncogenic stresses, including cell death [17,18]. With regard to cell death, ATF3 has previously been reported to be either an antiapoptotic or a proapoptotic regulator [18]. It seems that ATF3 alters gene expression and determines whether the FUdR-induced cell death occurs via necrosis or apoptosis [14].

Lamin-B1 is one of the nuclear lamins and a key structural component of the nuclear lamina, an intermediate filament meshwork that lies beneath the inner nuclear membrane [19]. The nuclear lamins are known to play a crucial role in fundamental cellular processes, including nuclear organization, chromatin segregation, DNA replication, and gene expression [19,20]. Interestingly, Freund et al. have reported that lamin-B1 decreases with cellular senescence [21]. Additionally, cytokeratin-19 is a type of cytoplasmic intermediate filament, constituting a key structural component of the cytoskeletal proteins [22,23]. Previously, several studies have indicated that the nuclear and cytoplasmic intermediate filament proteins, lamin-B1 and cytokeratin-19, are caspase substrates, which undergo caspase-mediated degradation during apoptosis [24,25,26]. Our finding suggests that high expressions of the nuclear and cytoplasmic intermediate filament proteins, lamin-B1 and cytokeratin-19, are important in necrosis, and decreased expressions of these proteins lead to apoptosis. We consider that a decrease in both nuclear and cytoplasmic intermediate filaments increases flexibility of the nucleus and cell structure, thereby leading to apoptosis [15].

The heat shock protein (HSP) family and related genes exhibited differential expression patterns in F28-7 and F28-7-A cells by comprehensive transcriptomic and/or proteomic analysis. These genes, including *Hsp110*, *Hsp70*, *Dnajb4*, and *Ahsa1*, are involved in the HSP90 chaperone complex. In particular, these analyses revealed that the activator of 90-kDa HSP ATPase homolog 1 (AHA1) is expressed at lower levels in F28-7 cells than in F28-7-A cells [13]. AHA1 is an activator of the HSP90 ATPase activity, which plays a key role in the regulation of ATP-dependent HSP90 chaperone activity [27,28,29,30]. HSP90 is one of the more abundant HSPs, and because it regulates the stability and function of a unique complement of signal transduction proteins, this chaperone is involved in a variety of important biological processes, including hormone signaling, cell cycle control, development, and cell death [31,32,33,34,35,36,37]. The HSP90 chaperone complex is driven by ATP binding and hydrolysis [38,39,40]. Its activity is regulated by cochaperones, such as AHA1, HSP72, cell division cycle 37, HSP90 cochaperone (CDC37), p23, carboxyl terminus of heat shock cognate (Hsc)70-interacting protein (CHIP), and immunophilins [41,42]. Therefore, it may be expected that HSP90 ATPase activity differs between F28-7 and F28-7-A cells. To investigate the involvement of HSP90 in FUdR-induced necrosis and apoptosis, we utilized an HSP90 inhibitor, geldanamycin (GA). Notably, the functional inhibition of HSP90 in necrosis-fated F28-7 cells resulted in a shift from FUdR-induced necrosis to apoptosis; apoptotic cell morphology and oligonucleosome DNA fragmentation were induced by combination treatment with FUdR and GA in F28-7 cells [13]. Conversely, in F28-7-A cells, such oligonucleosomal DNA fragmentation was induced by treatment with either FUdR alone or the combination of GA and FUdR. We also demonstrated the release of cytochrome c in F28-7 cells upon treatment with the combination of GA and FUdR. As expected, the release of cytochrome c was not observed in F28-7 cells treated with GA or FUdR alone [13]. These observations, therefore, suggest that HSP90 or an HSP90 client protein participates in the execution of necrotic cell death. We suspect that HSP90 functionality may be closely related to the expression of the identified cell death regulators: lamin-B1, cytokeratin-19, and ATF3. To investigate the possible involvement of HSP90 expression of these proteins, we used the HSP90-specific inhibitor GA [13]. In necrosis-fated F28-7 cells, the expression of these cell death regulators was decreased upon combination treatment with FUdR and GA. We conclude that the expression or stability of these cell death regulators may be mediated by HSP90 function.

Recently, we focused on the role of microRNAs (miRNAs), i.e., 21–25 nucleotides-long, endogenous, small noncoding RNAs, in the switch of cell death mechanism [43,44,45,46]. miRNAs can function as gene silencers by binding to the 3′-untranslated region (UTR) of target mRNAs, thereby inhibiting the initiation of protein synthesis and/or promoting mRNA cleavage [47,48]. We investigated miRNA expression profiles in necrosis-fated F28-7 and apoptosis-fated F28-7-A cells using miRNA microarrays. Previously, we identified several differentially expressed miRNAs, e.g., miR-351-5p and miR-743a-3p, in these cell models [49]. To determine whether the inhibition/overexpression of these candidate miRNAs modulate FUdR-induced cell death, we transfected the inhibitors of these miRNAs and/or synthetic miRNA mimics [49]. We found that higher expression of miR-351-5p in F28-7 cells upon transfection of the synthetic miR-351-5p mimic induced a shift from necrotic to apoptotic cell death. We confirmed via Western blot analysis that leakage of a known marker of necrotic cell death, high-mobility group box 1 (HMGB1) [50], from the nucleus into the culture medium in FUdR-induced necrosis was nearly eradicated by the transfection of miR-351 mimic. F28-7-A cells transfected with a miR-351 inhibitor underwent typical necrosis following treatment with FUdR; thus, the inhibition of miR-351 resulted in the shift from FUdR-induced apoptosis to necrosis in the apoptosis-fated F28-7-A cells.

We previously identified three new regulators of the mode of FUdR-induced cell death: the nuclear scaffold protein lamin-B1, the cytoplasmic scaffold protein cytokeratin-19, and transcription factor ATF3. To determine if increased miR-351 expression in F28-7 could modulate the expression levels of these three regulators, we investigated the protein levels of lamin-B1, cytokeratin-19, and ATF3 via Western blot analysis. The transfection of the miR-351-5p mimic in F28-7 cells led to a reduction in lamin-B1 protein, but not in cytokeratin-19 or ATF3. Notably, we also discovered that miR-351-5p directly interacted with lamin-B1 mRNA in a cell-free miRNA to mRNA binding evaluation system [51]. These findings suggest that miR-351-5p regulates nuclear scaffold lamin-B1 expression to mediate FUdR-induced apoptosis.

**Figure 2:**
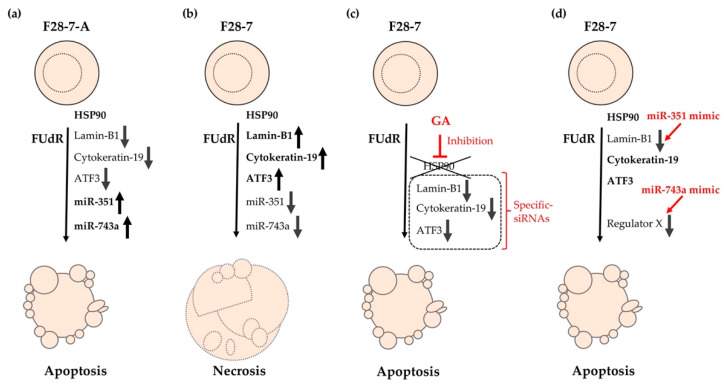
Cell death models of FUdR-induced necrosis and apoptosis in F28-7 and F28-7-A cells. (**a**) Treatment of F28-7-A cells with FUdR induces apoptosis. (**b**) Treatment of F28-7 cells with FUdR induces necrosis. (**c**) Co-treatment of the HSP90 inhibitor geldanamycin (GA) inhibits the expression of the switch regulators lamin-B1, cytokeratin-19, and activating transcription factor 3 (ATF3), thus resulting in apoptosis. Knockdown of switch regulators in F28-7 cells by small interfering RNAs (siRNAs) also causes a shift from FUdR-induced necrosis to apoptosis. (**d**) Increased expression of miR-351 or miR-743a in F28-7 cells by miR transfection mimics inhibits the expression of cell death regulator lamin-B1 or regulator X (unknown regulator), resulting in apoptosis. miR-351, miR-351-5p; miR-743a, and miR-743a-3p.

Furthermore, this phenotypic screening using miRNA mimics revealed that higher expression of miR-743a-3p in F28-7 cells via transfection of the synthetic miR-743a-3p mimic caused a shift from FUdR-induced necrosis to apoptosis. These findings suggest that the expression of miR-743a-3p plays a key role in FUdR-induced apoptosis. Further studies will be required to further elucidate the relationship between miR-743a-3p expression and the two modes of cell death: necrosis and apoptosis. The cell death models of FUdR-induced necrosis and apoptosis in F28-7 and F28-7-A cells are presented in Figure 2. In addition, Table 2 summarizes the characteristics of our identified cell death-switching regulators discovered in these cell death models.

## 4. Anticancer Strategy Targeting Cell Death Regulators of Necrotic to Apoptotic Cell Death

**Figure 3:**
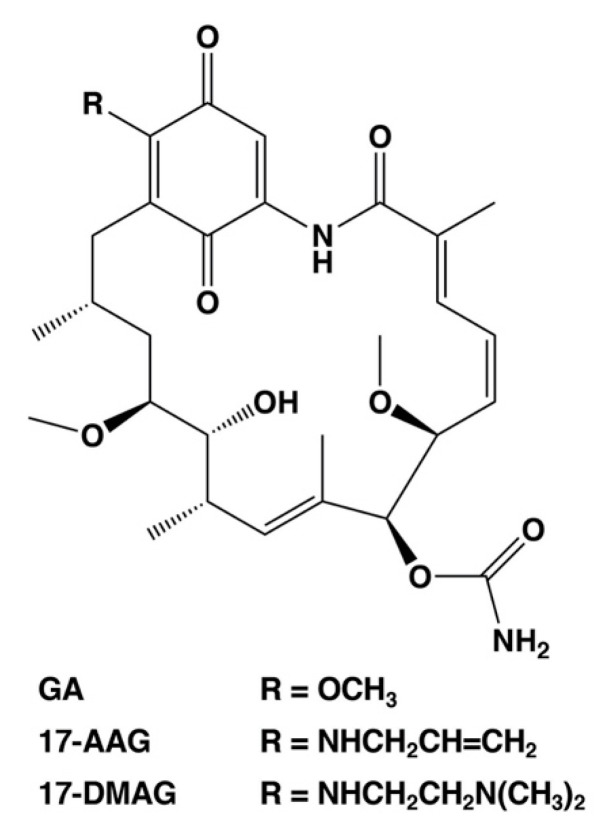
Chemical structures of selected HSP90 inhibitors. GA, geldanamycin; 17-AAG, 17-allylamino-17-demethoxy-geldanamycin (tanespimycin); 17-DMAG, 17-dimethylaminoethylamino-17-demethoxy-geldanamycin (alvespimycin).

As described above, the cell death regulators of the switch between necrosis and apoptosis in cancer cells could be identified using this cell death model. Necrosis causes hemorrhage and edema in tumor tissue, which are also side effects of anticancer medicine and radiation treatment [6,7,8]. In addition, necrotic cell death at the core of the tumor microenvironment promotes resistance to anticancer drugs and radiotherapy [6,7,8]. Moreover, it can protect cancer stem cells. Therefore, we consider the cell death switching from necrosis to apoptosis in cancer cells as a promising anticancer strategy. We suggest that HSP90, which regulates the expression of cell death regulators lamin-B1, cytokeratin-19, and ATF3, plays a significant role in the regulation of tumor necrosis. HSP90 is a potential anticancer drug target, and numerous drug candidates have been developed to inhibit its function [52,53,54], including GA analogs tanespimycin (17-AAG, 17-allylamino-17-demethoxy-geldanamycin) and alvespimycin (17-DMAG, 17-dimethylaminoethylamino-17-demethoxy-geldanamycin) [52,53]. These first line inhibitors have entered clinical trials for antitumor chemotherapy. Figure 3 shows the chemical structures of benzoquinone ansamycin HSP90 inhibitors, GA, 17-AAG, and 17-DMAG. These GA analog HSP90 inhibitors, tanespimycin and alvespimycin, have been reported to enhance binding affinity and anticancer efficacy as compared to GA [52,54,55,56]. In particular, tanespimycin represents one of the most studied HSP90 inhibitors with anticancer effects in various cancer cell models and in several clinical trials. In addition, other synthesized, small molecule inhibitors of HSP90, i.e., IPI-493 (tanespimycin metabolite), IPI-504 (retaspimycin hydrochloride), NVP-AUY922, AT13387, XL888, BIIB021, and PU-H71, have been studied by various clinical trials [52,54]. However, these HSP90 inhibitors demonstrated only moderate therapeutic efficacy when used in monotherapy [52,54]. Several studies have previously indicated that the HSP90 inhibitors are more effective in combination with several conventional therapies, i.e., chemotherapy, immunotherapy, and radiotherapy in experimental and clinical research [52,54].

Several previous studies have reported the important roles of HSP90 in TNF-induced necrosis and necroptosis. Inhibition of HSP90 function resulted in the degradation of death domain kinase, receptor-interacting protein (RIP), and the blockage of TNF-induced nuclear factor-κB activation [33]. Berghe et al. reported that inhibition of HSP90 function reverts TNF-induced necrosis to apoptosis [57], suggesting that HSP90 is crucial for cells to undergo necrotic cell death. In addition, Li et al. demonstrated that HSP90 and CDC37 cochaperone complex is required for RIP3 activation and the induction of TNF-α induced necroptosis [58]. Indeed, HSP90 inhibitors, GA, 17-AAG, and 17-DMAG, efficiently block TNF-α induced necroptosis by preventing RIP3 activation [58]. Furthermore, the activity of HSP90 modulates the stability of mixed lineage kinase domain-like protein and its oligomerization and translocation to the plasma membrane in TNF-induced necroptosis [59,60]. Interestingly, the disruption of HSP90 function prevents TNF-induced necroptosis in several models [58,59,60]. Notably, Li et al. reported that the administration of the HSP90 inhibitor 17-DMAG suppresses TNF-α induced systemic inflammatory response syndrome in a rat model [58]. These findings indicate the potential of HSP90 inhibitors as therapeutics for necrosis and necroptosis-related diseases. We propose that HSP90 inhibitors as adjuvants have the potential to effectively utilize the anticancer strategy of targeting the necrosis to apoptosis switching mechanism in the necrotic tumor microenvironment.

Moreover, we consider that miRNA can potentially control the switch from necrosis to apoptosis tumors. We identified miR-351-5p and miR-743a-3p as cell death regulators of necrosis and apoptosis [49]. In addition, we revealed that several miRNAs were dramatically altered in necrosis and apoptosis using these cell death models [49]. miRNA can modulate the post-transcriptional expression of dozens to hundreds of target genes, and miRNAs have been reported to be associated with various diseases, including cancer [43,44,45]. In the cancer research area, several miRNAs are used as tumor biomarkers and are being developed as targets for nucleic acid therapies [61,62]. We propose that necrosis- and apoptosis-regulated miRNAs are potentially effective targets of the unique anticancer strategy described.

## 5. Conclusions and Future Directions

We described the identification of five new regulators of cell death using a novel cell death model: the molecular chaperone HSP90, the nuclear scaffold protein lamin-B1, the cytoplasmic scaffold protein cytokeratin-19, the transcription factor ATF3, and the miRNAs miR-351-5p and miR-743a-3p. We further investigated the gene mutation of cell death regulators in necrosis and apoptosis via whole-exome sequencing analysis. In addition, we studied the whole leakage proteins, namely, secretome, of FUdR-induced necrosis and apoptosis via a shotgun proteomic approach. In the future, we will continue to elucidate the entire mechanism of switching between the modes of necrosis and apoptosis in human normal and cancer cells. These findings may lead to the development of new anticancer drugs that target cell death regulators, e.g., HSP90, lamin-B1, cytokeratin-19, ATF3, and miRNAs, which are involved in the switching mechanism from necrosis to apoptosis.

## Figures and Tables

**Table 1:** The biological and morphological features of FUdR-induced cell death.

Cell Line	F28-7 Cells	F28-7-A Cells
FUdR (EC_50_, nM)	1	1
Cell death mode	Necrosis	Apoptosis
Cell death morphological features	Swelling (Cell and organelles)	Membrane blebbing, shrinking (Cell and organelles)
DNA fragmentation	Chromosome size (100–200 kbp)	Oligonucleosome size
Mitochondrial events		
Membrane potential	Down	Down
Cytochrome c release	-	+
Cell death markers		
Caspase-3	Cleaved	Cleaved
PARP	Cleaved	Cleaved
Extracellular HMGB1	+	−

**Table 2:** Identification of switch regulators, necrosis, and apoptosis.

Name	Expression	Cell Death	Experiments	Observation	Ref
HSP90	NC	N > A	IH(GA)	MF, DL	[13]
Lamin-B1	High F28-7	N > A	KD(siR)	MF	[15,16]
Cytokeratin-19	High F28-7	N > A	KD(siR)	MF	[15]
ATF3	High F28-7	N > A	KD(siR)	MF	[14]
miR-351-5p	High F28-7-A	N > A/A > N	OE(miRm)/IH(miRi)	MF, HR	[49,51]
miR-743a-3p	High F28-7-A	N > A	OE(miRm)	MF	[49]

**Note:** NC, no change; N>A, necrosis to apoptosis; A > N, apoptosis to necrosis; IH, inhibition; GA, geldanamycin; KD, knockdown; siR, siRNA; OE, overexpression; miRm, microRNA mimic; miRi, microRNA inhibitor; MF, morphological feature; DL, DNA ladder; HR, high-mobility group box 1 (HMGB1) release; Ref, Reference.

## Abbreviations

ATF3	activating transcription factor 3
FUdR	floxuridine
GA	geldanamycin
HSP	heat shock protein
miRNA	microRNA
miR	miRNA
siRNA	small interfering RNA
TNF	tumor necrosis factor

## References

[1] Nicotera P., Melino G. (2004). Regulation of the apoptosis–necrosis switch. Oncogene.

[2] Galluzzi L., Vitale I., Abrams J.M., Alnemri E.S., Baehrecke E.H., Blagosklonny M.V., Dawson T.M., Dawson V.L., El-Deiry W.S., Fulda S. (2011). Molecular definitions of cell death subroutines: Recommendations of the Nomenclature Committee on Cell Death 2012. Cell Death Differ..

[3] Galluzzi L., Pedro J.M.B.-S., Vitale I., A Aaronson S., Abrams J.M., Adam D., Alnemri E.S., Altucci L., Andrews D.W., Annicchiarico-Petruzzelli M. (2014). Essential versus accessory aspects of cell death: Recommendations of the NCCD 2015. Cell Death Differ..

[4] Galluzzi L., Vitale I., Aaronson S.A., Abrams J.M., Adam D., Agostinis P., Alnemri E.S., Altucci L., Amelio I., Andrews D.W. (2018). Molecular mechanisms of cell death: Recommendations of the Nomenclature Committee on Cell Death 2018. Cell Death Differ..

[5] Tang D., Kang R., Berghe T.V., Vandenabeele P., Kroemer G. (2019). The molecular machinery of regulated cell death. Cell Res..

[6] Bredholt G., Mannelqvist M., Stefansson I.M., Birkeland E., Bø T.H., Øyan A.M., Trovik J., Kalland K.-H., Jonassen I., Salvesen H.B. (2015). Tumor necrosis is an important hallmark of aggressive endometrial cancer and associates with hypoxia, angiogenesis and inflammation responses. Oncotarget.

[7] Vellayappan B., Tan C.L., Yong C., Khor L.K., Koh W.Y., Yeo T.T., Detsky J., Lo S., Sahgal A. (2018). Diagnosis and Management of Radiation Necrosis in Patients With Brain Metastases. Front. Oncol..

[8] Karsch-Bluman A., Feiglin A., Arbib E., Stern T., Shoval H., Schwob O., Berger M., Benny O. (2018). Tissue necrosis and its role in cancer progression. Oncogene.

[9] Kakutani T., Ebara Y., Kanja K., Hidaka M., Matsumoto Y., Nagano A., Wataya Y. (1998). Different Modes of Cell Death Induced by 5-Fluoro-2′-deoxyuridine in Two Clones of the Mouse Mammary Tumor FM3A Cell Line. Biochem. Biophys. Res. Commun..

[10] Santi D.V., Peña V.A., Lam S.S. (1976). On the structure of the cofactor in the complex formed with thymidylate synthetase, 5,10-methylenetetrahydrofolate and 5-fluoro-2′-deoxyuridylate. Biochim. Biophys. Acta (BBA) Enzym..

[11] Yoshioka A., Tanaka S., Hiraoka O., Koyama Y., Hirota Y., Ayusawa D., Seno T., Garrett C., Wataya Y. (1987). Deoxyribonucleoside triphosphate imbalance. 5-Fluorodeoxyuridine-induced DNA double strand breaks in mouse FM3A cells and the mechanism of cell death. J. Boil. Chem..

[12] Kakutani T., Ebara Y., Kanja K., Takahashi K., Wataya Y. (1998). Activation of c-junand c-fosGenes in dNTP Imbalance Cell Death Induced With 5-Fluoro-2′-Deoxyuridine in Mouse Mammary Tumor FM3A Cell Line. Nucleosides Nucleotides.

[13] Sato A., Hiramoto A., Uchikubo Y., Miyazaki E., Satake A., Naito T., Hiraoka O., Miyake T., Kim H.-S., Wataya Y. (2008). Gene expression profiles of necrosis and apoptosis induced by 5-fluoro-2′-deoxyuridine. Genomics.

[14] Sato A., Nakama K., Watanabe H., Satake A., Yamamoto A., Omi T., Hiramoto A., Masutani M., Wataya Y., Kim H.-S. (2014). Role of activating transcription factor 3 protein ATF3 in necrosis and apoptosis induced by 5-fluoro-2?-deoxyuridine. FEBS J..

[15] Sato A., Satake A., Hiramoto A., Wataya Y., Kim H.-S. (2010). Protein Expression Profiles of Necrosis and Apoptosis Induced by 5-Fluoro-2′-deoxyuridine in Mouse Cancer Cells. J. Proteome Res..

[16] Sato A., Hiramoto A., Satake A., Miyazaki E., Naito T., Wataya Y., Kim H.-S. (2008). Association of Nuclear Membrane Protein Lamin B1 with Necrosis and Apoptosis in Cell Death Induced by 5-Fluoro-2′-Deoxyuridine. Nucleosides Nucleotides Nucleic Acids.

[17] Hai T., Hartman M.G. (2001). The molecular biology and nomenclature of the activating transcription factor/cAMP responsive element binding family of transcription factors: Activating transcription factor proteins and homeostasis. Gene.

[18] Thompson M.R., Xu D., Williams B.R. (2009). ATF3 transcription factor and its emerging roles in immunity and cancer. J. Mol. Med..

[19] Cohen M., Gruenbaum Y., Lee K.K., Wilson K.L. (2001). Transcriptional repression, apoptosis, human disease and the functional evolution of the nuclear lamina. Trends Biochem. Sci..

[20] Hutchison C.J. (2002). Lamins: Building blocks or regulators of gene expression?. Nat. Rev. Mol. Cell Boil..

[21] Freund A., Laberge R.-M., DeMaria M., Campisi J. (2012). Lamin B1 loss is a senescence-associated biomarker. Mol. Boil. Cell.

[22] Wu Y.-J., Rheinwald J.G. (1981). A new small (40 kd) keratin filament protein made by some cultured human squamous cell carcinomas. Cell.

[23] Moll R., Von Bassewitz D.B., Schulz U., Franke W.W. (1982). An Unusual Type of Cytokeratin Filament in Cells of a Human Cloacogenic Carcinoma Derived from the Anorectal Transition Zone. Differentiation.

[24] Ku N.-O., Omary M.B. (2001). Effect of Mutation and Phosphorylation of Type I Keratins on Their Caspase-mediated Degradation. J. Boil. Chem..

[25] Oshima R.G. (2002). Apoptosis and keratin intermediate filaments. Cell Death Differ..

[26] Kawahara A., Enari M., Talanian R.V., Wong W.W., Nagata S. (1998). Fas-induced DNA fragmentation and proteolysis of nuclear proteins. Genes Cells.

[27] Panaretou B., Siligardi G., Meyer P., Maloney A., Sullivan J.K., Singh S., Millson S.H., Clarke P.A., Naaby-Hansen S., Stein R. (2002). Activation of the ATPase Activity of Hsp90 by the Stress-Regulated Cochaperone Aha1. Mol. Cell.

[28] Lotz G.P., Lin H., Harst A., Obermann W.M.J. (2003). Aha1 Binds to the Middle Domain of Hsp90, Contributes to Client Protein Activation, and Stimulates the ATPase Activity of the Molecular Chaperone. J. Boil. Chem..

[29] Meyer P. (2004). Structural basis for recruitment of the ATPase activator Aha1 to the Hsp90 chaperone machinery. EMBO J..

[30] Siligardi G., Hu B., Panaretou B., Piper P.W., Pearl L.H., Prodromou C. (2004). Co-chaperone Regulation of Conformational Switching in the Hsp90 ATPase Cycle. J. Boil. Chem..

[31] Scheibel T., Buchner J. (1998). The Hsp90 complex—A super-chaperone machine as a novel drug target. Biochem. Pharmacol..

[32] Rutherford S.L., Lindquist S. (1998). Hsp90 as a capacitor for morphological evolution. Nature.

[33] Lewis J., Devin A., Miller A., Lin Y., Rodriguez Y., Neckers L., Liu Z.-G. (2000). Disruption of Hsp90 Function Results in Degradation of the Death Domain Kinase, Receptor-interacting Protein (RIP), and Blockage of Tumor Necrosis Factor-induced Nuclear Factor-κB Activation. J. Boil. Chem..

[34] Powers M.V., Workman P. (2006). Targeting of multiple signalling pathways by heat shock protein 90 molecular chaperone inhibitors. Endocr.-Relat. Cancer.

[35] Zhao C., Wang E. (2004). Heat shock protein 90 suppresses tumor necrosis factor alpha induced apoptosis by preventing the cleavage of Bid in NIH3T3 fibroblasts. Cell. Signal..

[36] Berghe T.V., Van Loo G., Saelens X., Van Gurp M., Brouckaert G., Kalai M., Declercq W., Vandenabeele P. (2003). Differential Signaling to Apoptotic and Necrotic Cell Death by Fas-associated Death Domain Protein FADD. J. Boil. Chem..

[37] Hoter A., El-Sabban M., Naim H.Y. (2018). The HSP90 Family: Structure, Regulation, Function, and Implications in Health and Disease. Int. J. Mol. Sci..

[38] Grenert J.P., Johnson B.D., Toft D.O. (1999). The Importance of ATP Binding and Hydrolysis by Hsp90 in Formation and Function of Protein Heterocomplexes. J. Boil. Chem..

[39] Obermann W.M., Sondermann H., Russo A.A., Pavletich N.P., Hartl F.U. (1998). In Vivo Function of Hsp90 Is Dependent on ATP Binding and ATP Hydrolysis. J. Cell Boil..

[40] Panaretou B., Prodromou C., Roe S.M., O’Brien R., Ladbury J.E., Piper P.W., Pearl L.H. (1998). ATP binding and hydrolysis are essential to the function of the Hsp90 molecular chaperone in vivo. EMBO J..

[41] Connell P., Ballinger C.A., Jiang J., Wu Y., Thompson L.J., Höhfeld J., Patterson C. (2000). The co-chaperone CHIP regulates protein triage decisions mediated by heat-shock proteins. Nature.

[42] Pearl L.H., Prodromou C. (2006). Structure and Mechanism of the Hsp90 Molecular Chaperone Machinery. Annu. Rev. Biochem..

[43] Ambros V. (2004). The functions of animal microRNAs. Nature.

[44] Bartel B. (2004). MicroRNAs: Genomics, biogenesis, mechanism, and function. Cell.

[45] Bartel B. (2009). MicroRNAs: Target Recognition and Regulatory Functions. Cell.

[46] Xu P., Guo M., Hay B.A. (2004). MicroRNAs and the regulation of cell death. Trends Genet..

[47] Lytle J.R., Yario T.A., Steitz J.A. (2007). Target mRNAs are repressed as efficiently by microRNA-binding sites in the 5’ UTR as in the 3’ UTR. Proc. Natl. Acad. Sci. USA.

[48] Lee I., Ajay S.S., Yook J.I., Kim H.S., Hong S.H., Kim N.H., Han S., Chinnaiyan A.M., Athey B. (2009). New class of microRNA targets containing simultaneous 5’-UTR and 3’-UTR interaction sites. Genome Res..

[49] Sato A., Omi T., Yamamoto A., Satake A., Hiramoto A., Masutani M., Tanuma S.-I., Wataya Y., Kim H.-S. (2016). MicroRNA-351 Regulates Two-Types of Cell Death, Necrosis and Apoptosis, Induced by 5-fluoro-2’-deoxyuridine. PLoS ONE.

[50] Scaffidi P., Misteli T., Bianchi M. (2002). Release of chromatin protein HMGB1 by necrotic cells triggers inflammation. Nature.

[51] Sato A., Ogino Y., Shimotsuma A., Hiramoto A., Kim H.-S., Wataya Y. (2020). Direct interaction analysis of microRNA-351-5p and nuclear scaffold lamin B1 mRNA by the cell-free in vitro mRNA/miRNA binding evaluation system. Nucleosides Nucleotides Nucleic Acids.

[52] Neckers L.M., Workman P. (2012). Hsp90 molecular chaperone inhibitors: Are we there yet?. Clin. Cancer Res..

[53] Tatokoro M., Koga F., Yoshida S., Kihara K. (2015). Heat shock protein 90 targeting therapy: State of the art and future perspective. EXCLI J..

[54] Shevtsov M., Multhoff G., Mikhaylova E.R., Shibata A., Ekimova I.V., Margulis B.A. (2019). Combination of Anti-Cancer Drugs with Molecular Chaperone Inhibitors. Int. J. Mol. Sci..

[55] Roe S.M., Prodromou C., O’Brien R., Ladbury J.E., Piper P.W., Pearl L.H. (1999). Structural Basis for Inhibition of the Hsp90 Molecular Chaperone by the Antitumor Antibiotics Radicicol and Geldanamycin. J. Med. Chem..

[56] Kamal A., Thao L., Sensintaffar J., Zhang L., Boehm M.F., Fritz L.C., Burrows F. (2003). A high-affinity conformation of Hsp90 confers tumour selectivity on Hsp90 inhibitors. Nature.

[57] Berghe T.V., Kalai M., Van Loo G., Declercq W., Vandenabeele P. (2002). Disruption of HSP90 Function Reverts Tumor Necrosis Factor-induced Necrosis to Apoptosis. J. Boil. Chem..

[58] Li D., Xu T., Cao Y., Wang H., Li L., Chen S., Wang X., Shen Z. (2015). A cytosolic heat shock protein 90 and cochaperone CDC37 complex is required for RIP3 activation during necroptosis. Proc. Natl. Acad. Sci. USA.

[59] Jacobsen A.V., Lowes K.N., Tanzer M.C., Lucet I.S., Hildebrand J.M., Petrie E.J., Van Delft M.F., Liu Z., A Conos S., Zhang J.-G. (2016). HSP90 activity is required for MLKL oligomerisation and membrane translocation and the induction of necroptotic cell death. Cell Death Dis..

[60] Zhao X.M., Chen Z., Zhao J.B., Zhang P.P., Pu Y.F., Jiang S.H., Hou J.J., Cui Y.M., Jia X.L., Zhang S.Q. (2016). Hsp90 modulates the stability of MLKL and is required for TNF-induced necroptosis. Cell Death Dis..

[61] Peng Y., Croce C.M. (2016). The role of MicroRNAs in human cancer. Signal Transduct. Target. Ther..

[62] Rupaimoole R., Slack F.J. (2017). MicroRNA therapeutics: Towards a new era for the management of cancer and other diseases. Nat. Rev. Drug Discov..

